# Application of Gestational Blood Glucose Control During Perinatal Period in Parturients with Diabetes Mellitus: Meta-Analysis of Controlled Clinical Studies

**DOI:** 10.3389/fsurg.2022.893148

**Published:** 2022-07-15

**Authors:** Tingting Wang, Wei Zhang

**Affiliations:** Department of Obstetric, Affiliated Hangzhou First People's Hospital, Zhejiang University School of Medicine, Hangzhou, China

**Keywords:** gestational diabetes mellitus, glycemic control, insulin, oral hypoglycemic agents, meta-analysis

## Abstract

**Background:**

Gestational diabetes mellitus (GDM) is a common metabolic disorder. Hyperglycemia may cause gestational hypertension, increase the probability of infection, abnormal embryonic development, and increase the abortion rate. Oral hypoglycemic drugs may be another effective means of blood glucose control in addition to insulin injection. We included controlled clinical studies for meta-analysis to understand the effect of oral hypoglycemic drugs in gestational diabetes.

**Methods:**

The databases were searched with the keywords “*Glycemic control*” & “*gestational diabetes*”: Embase (January, 2000–August, 2021), Pubmed (January, 2000–August, 2021), Web of Science (January, 2000–August, 2021), Ovid (January, 2000–August, 2021), and *ClinicalTrials.org* to obtain the randomized controlled trial (RCT) literatures related to the treatment of gestational diabetes with oral hypoglycemic drugs, after screening, the *R* language toolkit was used for the analysis.

**Results:**

A total of 10 articles with a total of 1,938 patients were included, 7 studies used *metformin* as an hypoglycemic agent. Meta-analysis showed that oral *metformin* had no significant difference in fasting blood glucose levels after the intervention compared with insulin injection [*MD *= −0.35, 95%CI(−0.70,1.40), *Z *= 0.66, *P *= 0.51], with no significant difference in postprandial blood glucose levels after intervention [*MD *= −2.20, 95%CI(−5.94,1.55), *Z *= −1.15, *P *= 0.25], and no statistical difference in glycosylated hemoglobin [*MD *= 0.10, 95%CI(−0.17,−0.04), *Z *= −0.94, *P *= 0.31]. *Metformin* was more conducive to reducing maternal weight during pregnancy than insulin [*MD *= −1.55, 95%CI(−2.77,−0.34), *Z *= −2.5, *P *= 0.0123], *metformin* reduced the abortion rate compared with insulin [*RR *= 0.81, 95%CI(0.63,1.05), *Z *= −2.61, *P *= 0.015], and reduced cesarean section rate [*RR *= 0.66, 95%CI(0.49,0.90), *Z *= −3.95, *P *= 0.0001].

**Discussion:**

The application of oral hypoglycemic drug *metformin* in blood glucose control of gestational diabetes can play a hypoglycemic effect equivalent to insulin and can control the weight of pregnant women, reduce the rate of abortion and cesarean section, and improve pregnancy outcomes.

## Introduction

Gestational diabetes mellitus (GDM) is a common metabolic disorder that refers to varying degrees of abnormal glucose metabolism that occurs for the first time during pregnancy (1). It has been reported its incidence ranges from 1.32% to 3.75%. Gestational diabetes is specific, and the parturient has no history of diabetes before pregnancy (2). But due to a variety of physiological changes during pregnancy, the reabsorption of glucose by the renal tubules is weakened, so that the sugar content in the urine is high, which in turn can cause diabetes, which is gestational diabetes (3). The effect of gestational diabetes on maternal and fetal outcomes is related to the degree of glycemic control (4). Hyperglycemia may cause maternal gestational hypertension, increase the chance of infection, and may also cause abnormal embryonic development and increase the rate of miscarriage (5). Some patients can achieve the expected blood glucose range through lifestyle intervention, including changing lifestyle, reasonable diet, appropriate exercise, prevention of infection, and regular testing of blood glucose levels (6). However, some patients fail to reach the ideal blood glucose level and still need drugs for intervention (7). Injection of insulin is the most common method of blood glucose control. Besides, compared with insulin, that oral hypoglycemic agent is convenient to use and ideal for hypoglycemic effect. And the efficacy and safety of oral hypoglycemic drugs in GDM patients have been reported, but there is still a lack of systematic evaluation and comprehensive analysis in clinical practice (8). In order to understand the efficacy of oral hypoglycemic agents in the treatment of gestational diabetes, we included controlled clinical studies for meta-analysis to provide evidence for the clinical treatment of this disease.

## Method

### Inclusion of Studies

We followed the PICOS principle to develop inclusion criteria (P-participants, I-intervention, C-control, O-outcome, S-study type): (1) Study type: The literatures published after January, 2000 were limited to randomized controlled trials (RCTs), the language was English, and individual cases, guidelines, systematic analysis, and case-control studies of non-RCT studies were excluded. (2) Study subjects: The participants were pregnant women aged 18–45 years, 14–35 weeks of gestational age (GA), diagnosed with diabetes (we did not limit pregnant women to type 1 or type 2 diabetes), fasting blood glucose ≥7.0 mmol/L [126 mg/dl], and HbA1c ≥48 mmol/mol [≥6.5%] (9)). (3) Grouping and control: randomization must be taken in the study, we do not limit the randomization method (computer random number or manual random number), we do not limit the allocation concealment and blind method, but we will perform the quality assessment of the literature. (4) Intervention method: All patients were given routine prenatal care and iron, calcium, folic acid, and vitamin D supplementation after enrollment, all patients were given regulation from the diet and lifestyle, if the regulation failed (fasting blood glucose higher than 95 mg/dl and postprandial blood glucose higher than 40 mg/dl), the intervention was carried out. The control group was given conventional insulin injection, and the observation group was given hypoglycemic drugs (biguanides or Glinides). (5) Outcome indicators: The literature must provide observation indicators and statistical methods, provide outcome data, or indicate the accessible storage address of data.

### Literature Search Strategy

Search database: Embase (January, 2000–August, 2021), Pubmed (January, 2000–August, 2021), Web of Science (January, 2000–August, 2021), Ovid (January, 2000–August, 2021), and *ClinicalTrials.org*. The search method was keyword rapid search, and the input keywords were: “*Glycemic control*” and “*gestational diabetes*.”

### Selection of Literatures

*SCREEN* and inclusion of articles were done independently by two researchers, and in case of discrepancies during this process, a third person was consulted for agreement. After the initial search, we combined all retrieved articles with “*. Enw”* is reserved with suffix name and is managed uniformly after imported by Endnote X9 software. The software menu of “*References”* -> “*find duplicates”* allows the software to de duplication the retrieved literatures, and then browse the title and author of the literatures by manual method. For the literatures with a similar title and the same author, browse the abstract of the literatures. If the time, place, and number of participants of the study coincide, it is considered that the study is repeated. We only retain the literatures with the later publication time. By reading the title and abstract of the literature for preliminary screening, we remove the literature that obviously does not meet the inclusion requirements; for the remaining literature, we use the “*Find full text*” function of the software to obtain the full text of the literature. For some unobtainable literature, we search the database of the literature or the publication magazine to obtain the full text of the literature; if the literature cannot be obtained through the network, we try to contact the original author (Find via email) to obtain the original text; if it still fails, we exclude the literature. Literatures that were obtained were read and checked for completeness of literature data, and articles with missing data were excluded.

### Data Extraction

After obtaining the full text of the literature, we use the self-made data table to extract the data information in the literature. Include the following contents: (1) Basic data of the literature: publication time, author, and region; (2) Characteristics of the study subjects: patient age, race, BMI, family history of diabetes, whether the first pregnancy, hypertension during pregnancy, fasting blood glucose, blood glucose (breakfast, lunch, and dinner), and glycosylated hemoglobin (HbA1c); (3) Literature intervention methods: grouping method, number of participants in each group, grouping intervention method, intervention time, and follow-up time; (4) Outcome data.

### Outcome Indicators

Blood glucose control indicators: (a) fasting glycemia; (b) postprandial glycemia after lunch; (c) HbA1c postpartum;

Maternal situation and obstetric outcome indicators: (a) maternal weight gain; (b) abortion rate; (c) cesarean deliveries.

### Statistical Methods

We used *R* language development environment (R version 4.1.2 released by “The R foundation for statistical computing”) to summarize and analyze the data of multiple studies. We entered the key data into *CSV* files, read the data under *RGUI*, and used Meta tool of *RGUI* environment (metabin/metacont/metainf/metabias/funnel) to obtain the summary data of continuous variables and binary variables. *MD* (mean, difference) effect size was used for continuous variables, and *RR* (Risk Ratio) effect size was used for dichotomous variables, with 95%CI as the confidence interval, and *P *< 0.05 was considered statistically significant. For the heterogeneity among different studies, *I*^2^ test was used for the analysis and *Q* check. The heterogeneity was not statistically significant when *I^2 ^*< 50% or *P *≥ 0.1, that means there was no (or acceptable) heterogeneity among the literatures, otherwise it indicated that there was heterogeneity among the literatures; if there was no statistical heterogeneity among the literatures, the fixed-effect model was used; if there was heterogeneity, the random effect model was used; the analysis results were presented in forest plot; publication bias was reported in the funnel plot.

### Heterogeneity Investigation and Sensitivity Analysis

We try to analyze the heterogeneous literatures to determine the source of heterogeneity.

## Results

### Literature Screening Results

**Figure 1:**
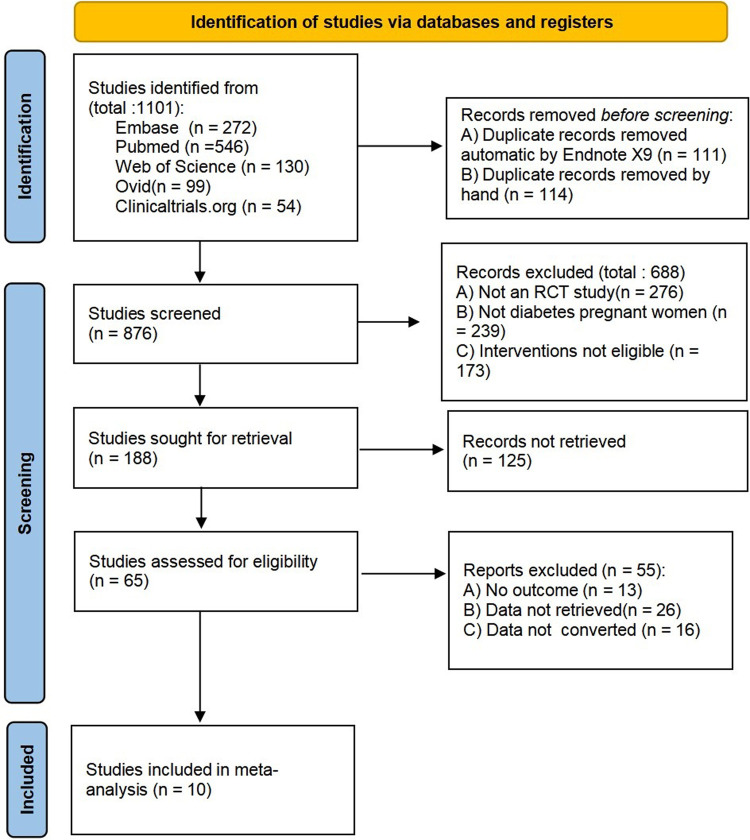
The selection flow chart.

In this search, 1,101 literatures were initially searched, 10 literatures (10–19) were finally included, 1,938 patients were included, and we listed three typical cases for exclusion: (a) the literature (20) was a retrospective observational study, so it was excluded; (b) the literature (21) was a pilot study, the number of patients included was too small, 14 cases in total; (c) the literature (22) was an observational study, without comparative data. The selection flowchart is shown in Figure 1.

### Basic Characteristics of Literatures

The published years of the studies included in this meta-analysis ranged from 2012 to 2021. The study subjects were all pregnant women with diabetes, aged 18–45 years. The minimum number of patients in the group was 32, and the maximum number was 253. Among them, three studies used *glyburide* as a hypoglycemic drug, while seven studies used *metformin* as a hypoglycemic drug, as shown in Table 1.

**Table 1:** Basic characteristics, intervention measures, follow-up time, and outcome indicators of the included literatures.

Author	Year of publication	Women age (years)	BMI (kg/m^2^)	Population (E/C)	Intervention group	Control group	Outcome indicators
Picón-César MJ et al. (10)	2021	34.86 ± 4.83	30.42 ± 5.42	100/100	Metformina Sandoz 850 mg/d, maximum 2,550 mg/d	Insulin 0.1 IU/kg/meal	(a) (b) (c) (d) (e) (f)
Kulshrestha V et al. (11)	2021	29.7 ± 4.4	25.5 ± 4.0	49/50	Metformin 1,000 mg twice daily	Insulin 0.1 IU/kg/meal	(d) (f)
Feig DS et al. (12)	2016	34.7 ± 5.0	35.0 ± 7.1	253/249	Metformin 1,000 mg twice daily	Insulin 0.1 IU/kg/meal	(c) (e) (f)
Casey BM et al. (13)	2015	31.3 ± 6	29.0 ± 4.8	189/186	Glyburide maximum of 20 mg per day	Insulin 0.1 IU/kg/meal	(d) (e) (f)
Beyuo T et al. (14)	2015	33.51 ± 4.67	33.47 ± 6.95	113/117	Metformin start dose 500 mg/d, max 2,500 mg/d	Insulin 0.1 IU/kg/meal	(a) (b)
Ainuddin J et al. (15)	2015	30.6 ± 2.9	N/A	43/75	Metformin start dose 500 mg/d, max 2,500 mg/d	Insulin 0.1 IU/kg/meal	(a) (b) (c) (d) (e)
Mirzamı M et al. (16)	2015	29.50 ± 4.06	N/A	37/59	1.25 mg glyburide with morning meal	Insulin 0.4 unit/kg	(a) (b)
Spaulonci CP et al. (17)	2013	31.93 ± 6.02	31.96 ± 4.75	47/47	Initial metformin dose of 1,700 mg/d (850 mg three times a day)	Insulin 0.4 unit/kg	(a) (b)
Tempe A et al. (18)	2013	N/A	N/A	32/32	Glyburide 2.5 mg orally as the initial dose	Insulin 0.4 unit/kg	(e) (f)
Niromanesh S et al. (19)	2012	30.7 ± 5.5	28.1 ± 4.0	80/80	initial metformin dose of 500 mg	Insulin 0.7 U/kg/d	(a) (b) (c) (d) (f)

*Abbreviation: E indicates the intervention group and C indicates the control group*.

*Outcomes:* (*a*) *Fasting blood glucose;* (*b*) *Postprandial glycemia after lunch;* (*c*) *HbA1c;* (*d*) *Maternal weight gain;* (*e*) *Abortion rate;* (*f*) *Cesarean section rate*.

### Bias Risk Assessment and Quality Evaluation of the Included Literatures

The use of Cochrane handbook for systematic reviews of interventions for risk of bias assessment in the included literature is shown in Table 2, all literatures had a detailed description for randomization and drop-out cases, without selective reporting of risk of bias and other risks. The literatures (10, 16) reported blindness, while the literatures (18) did not specify allocation concealment, which may cause selective risk.

**Table 2:** Risk of bias assessment and quality evaluation based on Cochrane Collaboration.

Study	Random sequence generation	Classification hiding	Blind method	Data integrity	Optional reporting	Other bias	Quality evaluation
Picón-César MJ et al. (10)	Low	Low	Unclear	Low	Low	Low	B
Kulshrestha V et al. (11)	Low	Low	Low	Low	Low	Low	A
Feig DS et al. (12)	Low	Low	Low	Low	Low	Low	A
Casey BM et al. (13)	Low	Low	Low	Low	Low	Low	A
Beyuo T et al. (14)	Low	Low	Low	Low	Low	Low	A
Ainuddin J et al. (15)	Low	Low	Low	Low	Low	Low	A
Mirzamı M et al. (16)	Low	Low	Unclear	Low	Low	Low	B
Spaulonci CP et al. (17)	Low	Low	Low	Low	Low	Low	A
Tempe A et al. (18)	Low	Unclear	Low	Low	Low	Low	B
Niromanesh S et al. (19)	Low	Low	Low	Low	Low	Low	A

### Meta-Analysis Results

#### Fasting Blood Glucose (mg/dl)

A total of six literatures (10, 14–17, 19) reported the fasting blood glucose of pregnant women after blood glucose control intervention, with heterogeneity between the literatures (*I*^2^*^ ^*= 67%, *P *< 0.01). The random effect mode combined analysis was used. There was no statistically significant difference in fasting blood glucose level after intervention between hypoglycemic drugs and insulin [*MD *= −0.67, 95%CI(−3.08,1.75), *Z *= 0.87, *P *= 0.25].

**Figure 2:**
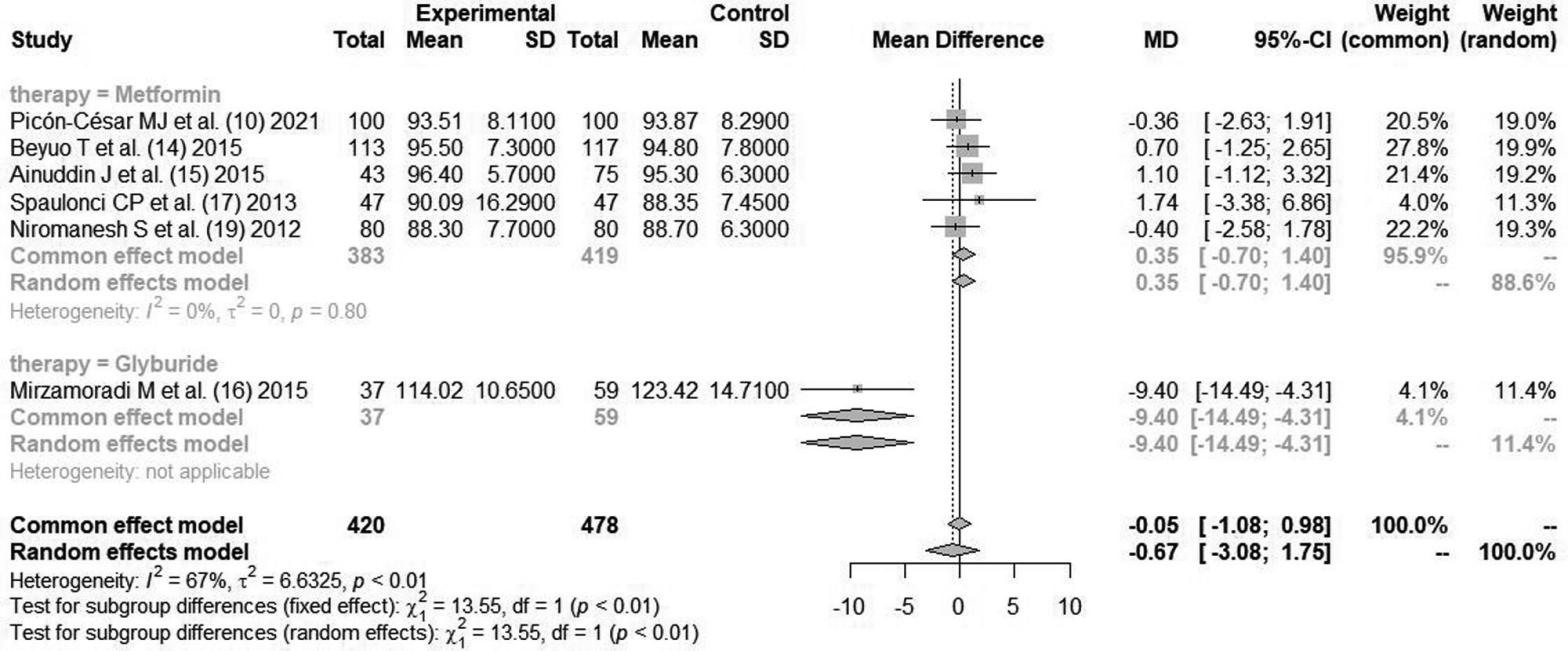
Comparison of fasting glycemia after glycemic control during pregnancy.

The study was further divided into two subgroups according to hypoglycemic drugs (*metformin* group and *glibenclamide* group). *Metformin* included five literatures. There was no statistically significant heterogeneity between the literatures (*I*^2^*^ ^*= 0%, *P *= 0.80). The pooled effect size for fasting blood glucose level after the intervention compared with insulin was [*MD *= −0.35, 95%CI(−0.70,1.40), *Z *= 0.66, *P *= 0.51]. The *glibenclamide* group contained only one article, and its effect size on fasting blood glucose compared with insulin was [*MD *= −9.40, 95%CI(−14.49,−4.31), *Z *= −3.62, *P *= 0.0003], as shown in Figure 2.

#### Postprandial Glycemia After Lunch (mg/dl)

A total of six literatures (10, 14–17, 19) reported the blood glucose content of pregnant women after lunch after blood glucose control intervention, with heterogeneity between the literatures (*I*^2^*^ ^*= 74%, *P *< 0.01). The random effects model combined analysis was used. There was no statistically significant difference in postprandial blood glucose level after intervention between hypoglycemic drugs and insulin [*MD *= −2.60, 95%CI(−5.75,0.56), *Z *= −1.61, *P *= 0.11].

**Figure 3:**
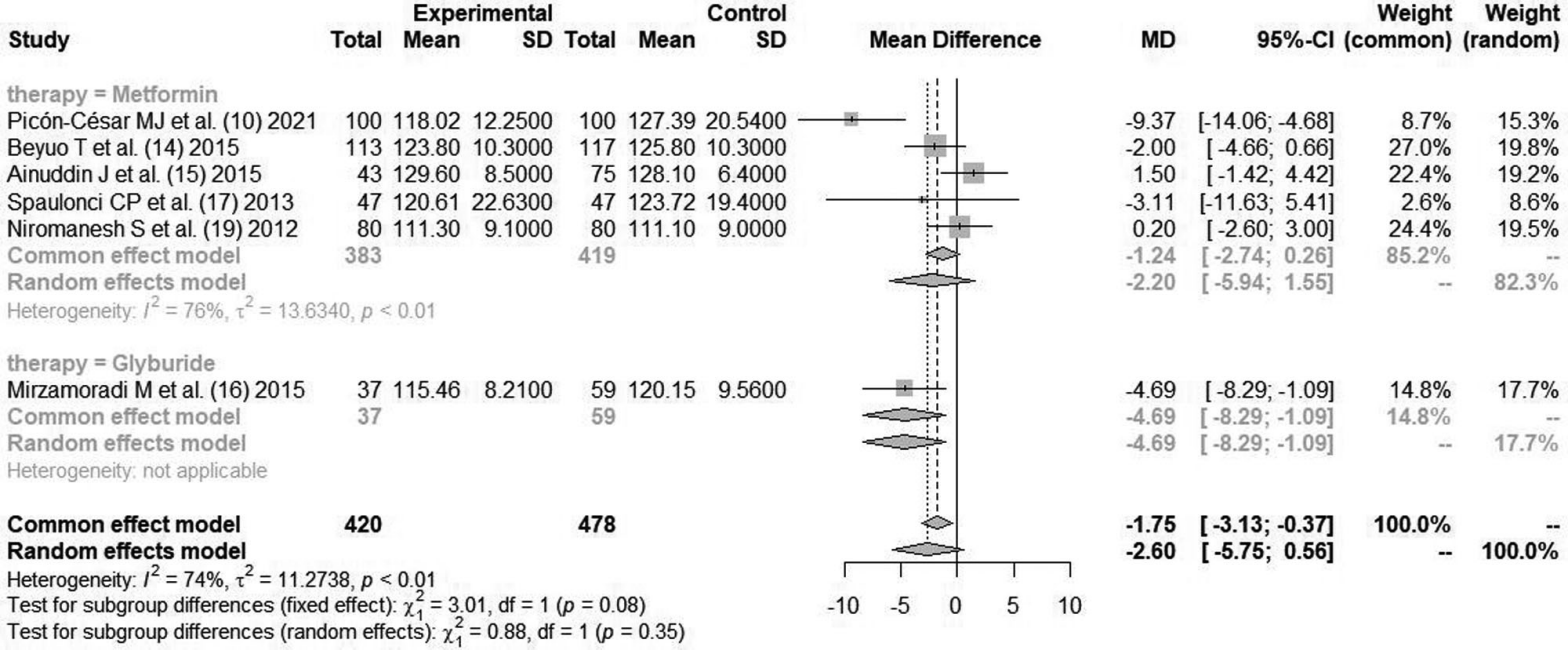
Comparison of postprandial glycemia after lunch during pregnancy.

The patients were further divided into two subgroups according to hypoglycemic drugs (*metformin* group and *glibenclamide* group). *Metformin* included five literatures. There was statistically significant heterogeneity between the literatures (*I*^2^*^ ^*= 76%, *P *< 0.01). The pooled effect size for postprandial blood glucose level after the intervention compared with insulin was [*MD *= −2.20, 95%CI(−5.94,1.55), *Z *= −1.15, *P *= 0.25]. There was only one article in the *glibenclamide* group, and the effect size on blood glucose compared with insulin was [*MD *= −4.69, 95%CI(−8.29,−1.09)], as shown in Figure 3.

#### Glycosylated Hemoglobin (HbA1c) (%)

**Figure 4:**
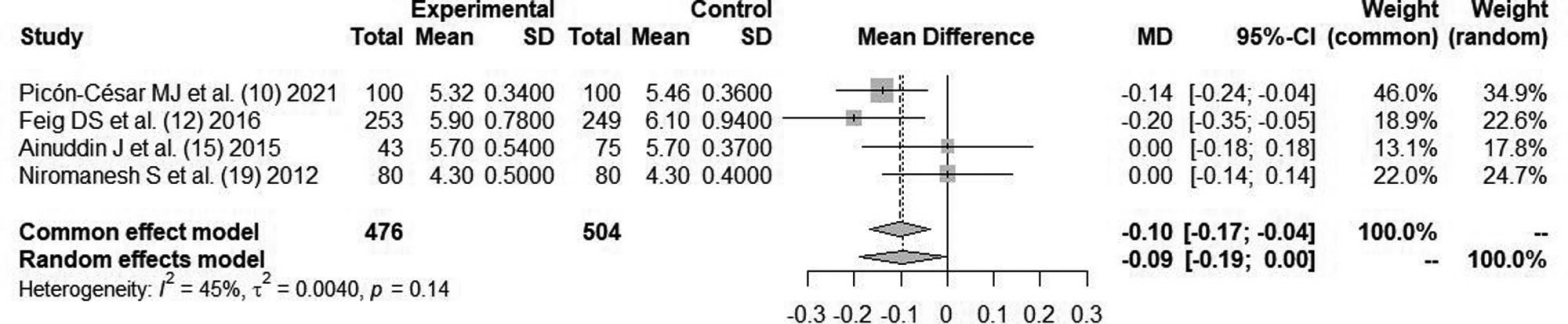
Comparison of postpartum glycosylated hemoglobin (HbA1c postpartum) after glycemic control during pregnancy.

A total of four literatures (10, 12, 15, 19) reported the changes of glycated hemoglobin index after blood glucose control. All studies used *metformin* as the hypoglycemic agent. Since there was no statistical heterogeneity between the literatures (*I*^2^*^ ^*= 45%, *P *= 0.14), the fixed-effect mode combined analysis was used. There was no statistical difference in glycosylated hemoglobin between *metformin* and insulin for blood glucose control [*MD *= 0.10, 95%CI(−0.17,−0.04), *Z *= −0.94, *P *= 0.31], as shown in Figure 4.

#### Maternal Weight Gain (kg)

**Figure 5:**
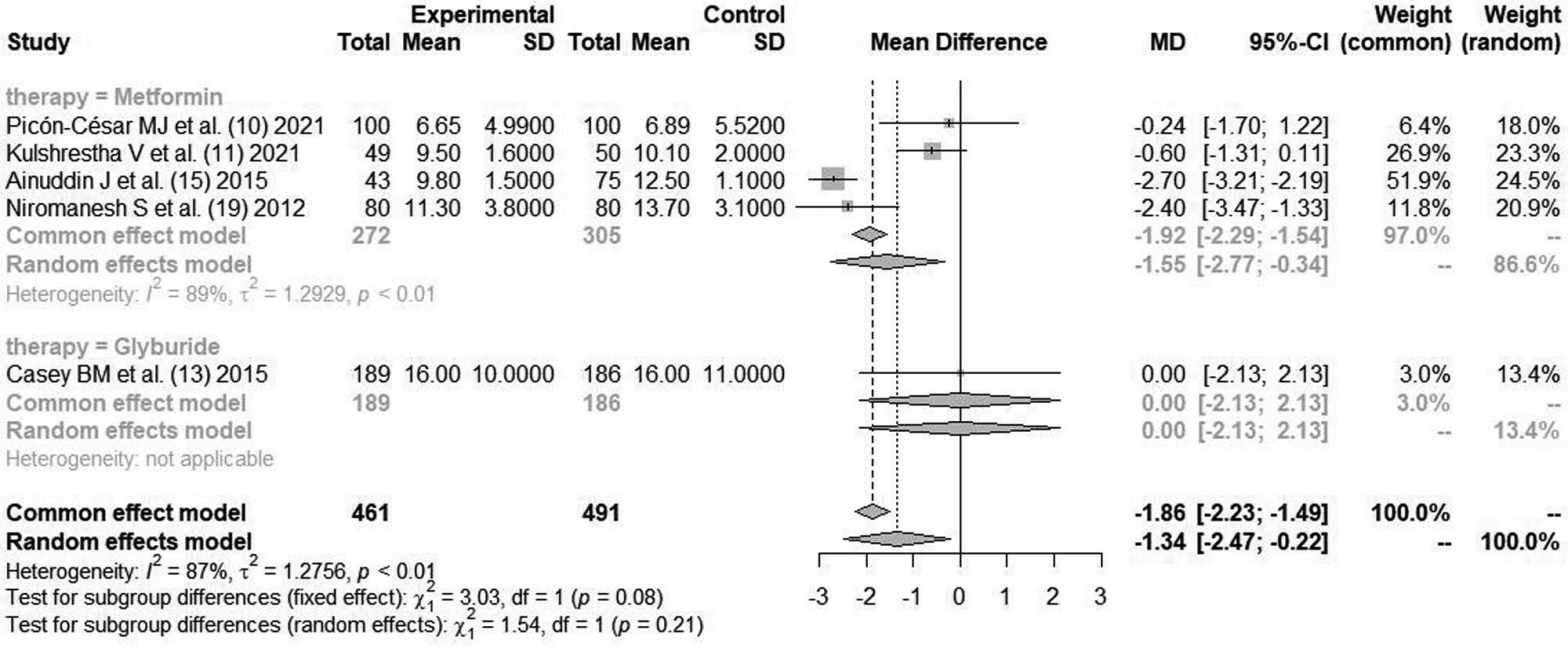
Comparison of maternal weight gain after blood glucose control during pregnancy.

Four literatures (10, 11, 15, 19) reported maternal weight gain indicators after glycemic control with *metformin*. Cause there was statistical heterogeneity between the literatures (*I*^2^*^ ^*= 87%, *P *< 0.01), the random effects model combined analysis was used. There was a statistical difference in maternal weight gain between *metformin* and insulin for glycemic control [*MD *= −1.55, 95%CI(−2.77,−0.34), *Z *= −2.5, *P *= 0.0123], as shown in Figure 5.

#### Abortion Rate

Three literatures (10, 12, 15) reported the indicators of maternal abortion rate after using *metformin* for blood glucose control. Cause there was no statistical heterogeneity between the literatures (*I*^2^*^ ^*= 8%, *P *= 0.34), the fixed effect mode combined analysis was used. There was statistical difference in maternal abortion rate between *metformin* and insulin for blood glucose control [*RR *= 0.81, 95%CI(0.63,1.05), *Z *= −2.61, *P *= 0.015].

**Figure 6:**
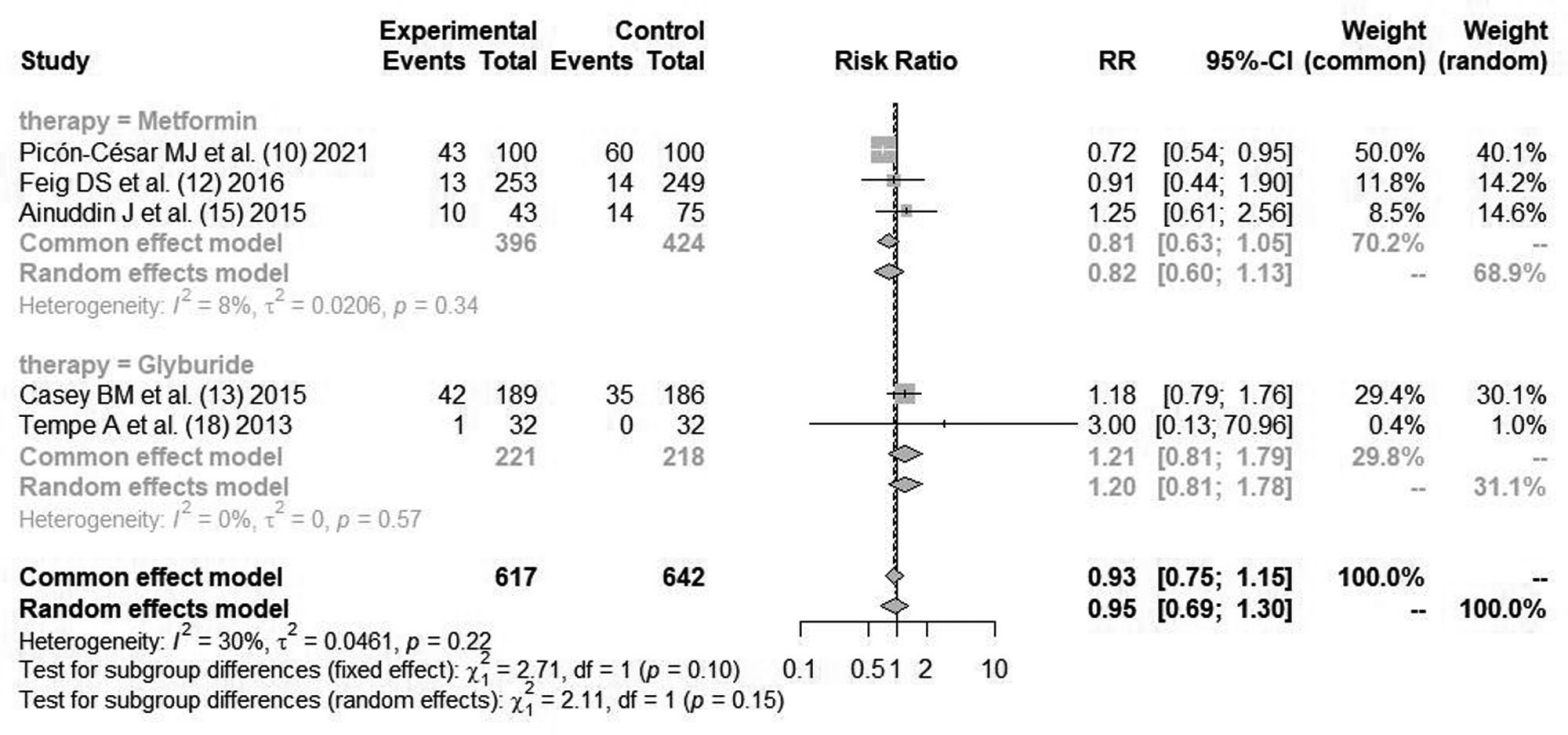
Comparison of maternal abortion rate after blood glucose control during pregnancy.

Two literatures (13, 18) reported the indicators of maternal abortion rate after using *glibenclamide* for blood glucose control. Cause there was no statistical heterogeneity between the literatures (*I*^2^*^ ^*= 0%, *P *= 0.57), the fixed effect mode combined analysis was used. There was no statistical difference in maternal abortion rate between *glibenclamide* and insulin for blood glucose control [*RR *= 1.21, 95%CI(0.81,1.79), *Z *= 0.93, *P *= 0.35], as shown in Figure 6.

#### Cesarean Section Rate

Three literatures (10, 11, 19) reported the indicators of cesarean section rate of parturients after glycemic control with *metformin*. Cause there was statistical heterogeneity between the literatures (*I*^2^*^ ^*= 50%, *P *= 0.14), the random effects model was used for combined analysis. There was statistical difference in cesarean section rate between *metformin* and insulin for glycemic control [*RR *= 0.66, 95%CI(0.49,0.90), *Z *= −3.95, *P *= 0.0001].

**Figure 7:**
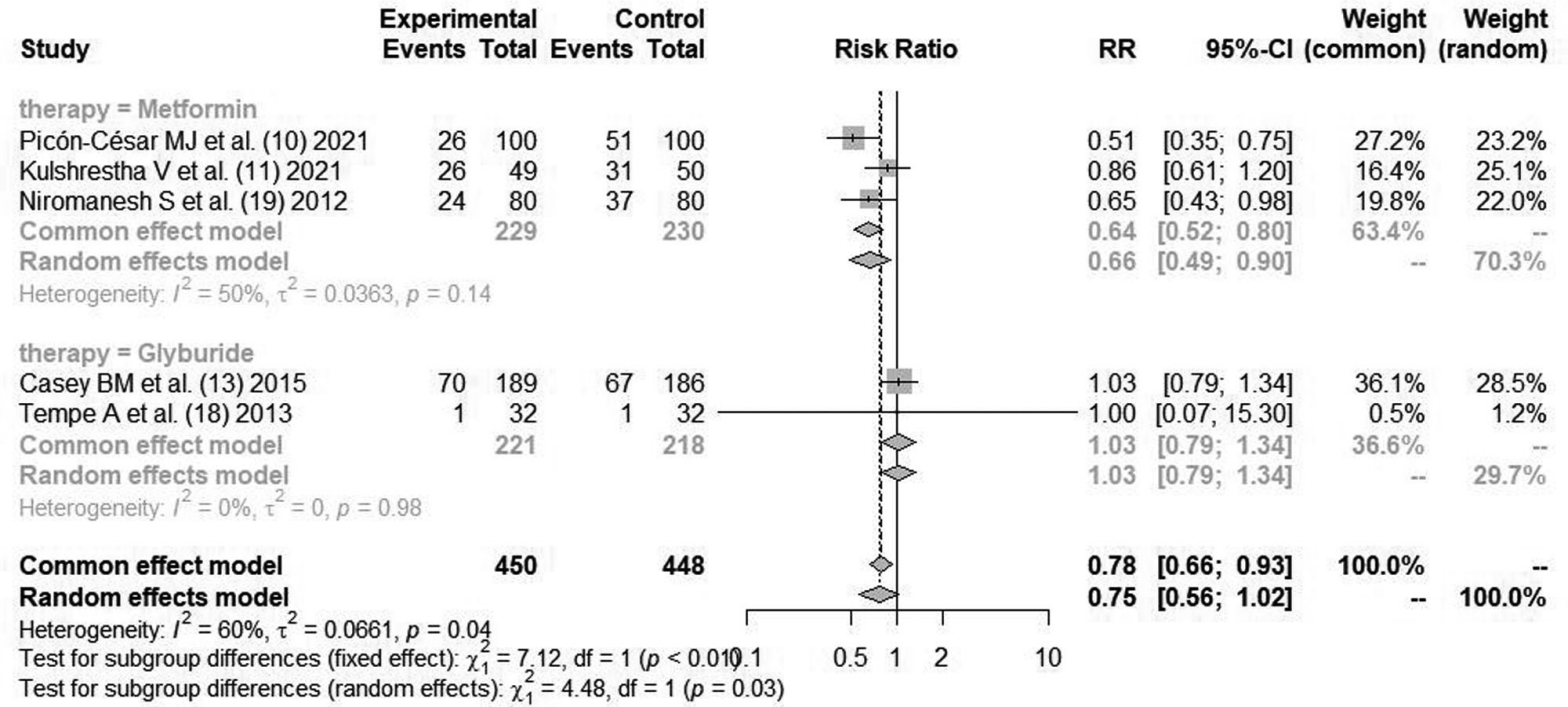
Comparison of cesarean section rate after blood glucose control during pregnancy.

Two literatures (13, 18) reported the indicators of maternal cesarean section rate after using *glibenclamide* for blood glucose control. Cause there was no statistical heterogeneity between the literatures (*I*^2^*^ ^*= 0%, *P *= 0.98), the fixed effect mode combined analysis was used. There was no statistical difference in maternal cesarean section rate between *glibenclamide* and insulin for blood glucose control [*RR *= 0.78, 95%CI(0.66,0.93), *Z *= 0.88, *P *= 0.44], as shown in Figure 7.

#### Heterogeneity Investigation and Sensitivity Analysis

In the analysis of fasting blood glucose, six articles (10, 14–17, 19) had heterogeneity (*I*^2^*^ ^*= 67%, *P *< 0.01), but after being divided into two subgroups according to glucose-controlling drugs, five articles within the *metformin* group had no heterogeneity (*I*^2^*^ ^*= 0%, *P *= 0.80), which suggested that glucose-controlling drugs were the greatest source of heterogeneity.

#### Analysis of Publication Bias

**Figure 8:**
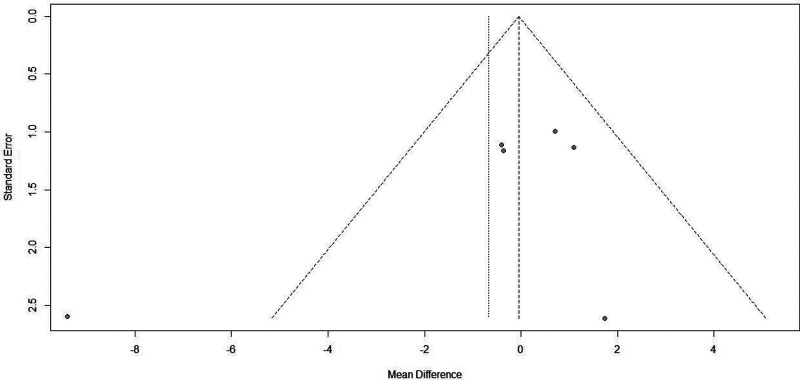
Funnel plot of fasting blood glucose indicators after blood glucose control during pregnancy.

In the analysis of fasting blood glucose, the funnel plot showed that the two sides were not evenly distributed, suggesting the presence of publication bias, as shown in Figure 8.

## Discussion

Ten RCTs with a total of 1,938 participants were included in this study, including seven studies using *metformin* as an oral hypoglycemic agent and three studies using *glibenclamide* as an oral hypoglycemic agent. The results of this study showed that the use of *metformin* as an oral hypoglycemic agent in gestational diabetes had no significant difference in glycemic control (fasting blood glucose, blood glucose, and glycosylated hemoglobin) compared with the utility of insulin injection, but the use of *metformin* could control maternal weight and improve pregnancy outcomes (reduce the rate of miscarriage and cesarean section). Both domestic and foreign guidelines recommend *metformin* as a first-line hypoglycemic drug. For patients with gestational diabetes, glucose control can be performed by intramuscular injection of insulin. *Metformin*, as a common hypoglycemic agent, promotes glucose uptake by target cells in the body, thereby regulating blood glucose levels (23). Studies (24) have revealed that *metformin* is mainly absorbed by the small intestine after oral administration, is not metabolized by the liver in the body, is mainly excreted unchanged by the kidney with the urine, and *metformin* itself has no hepatorenal toxicity and can be used normally in patients with normal liver and kidney function, so it has no negative impact on maternal and fetal outcomes. In addition, *metformin* belongs to the biguanide class of hypoglycemic agents, which control blood glucose by oral administration and can improve insulin therapy by improving insulin sensitivity, so it can be used in combination with insulin to better control blood glucose (25). But it is worth noting that during insulin therapy, the dose needs to be continuously adjusted, otherwise it will lead to hypoglycemic symptoms in patients, whether the blood glucose level is too high or too low, which will affect the safety of mothers and infants (26).

*Glibenclamide* is the second generation of sulfonylurea long-acting secretagogue, which produces the hypoglycemic effect by stimulating insulin cells to release insulin. It is suitable for mild and moderate non-insulin-dependent diabetes mellitus with unsatisfactory efficacy when diet is used alone. The results of the literature showed that the *glibenclamide* used as a glucose-controlling drug during pregnancy was superior to insulin therapy in lowering fasting blood glucose, but the evidence was insufficient cause too few articles were included. In a study by Moore LE et al. (27), *metformin* was compared with *glibenclamide* in gestational diabetes and found to have a 2.1-fold higher rate of glucose control failure with *metformin* than with *glibenclamide*. The efficacy and safety of *glibenclamide* remain to be deeply explored by more RCT studies.

The results of the study by Ashoush S et al. (28) showed that *metformin* in combination with insulin may be a better option for some patients whose glycemic control cannot be achieved with *metformin*. Literature (15) counted the cost of oral hypoglycemic agents using *metformin* throughout pregnancy, which was 4.02 ± 1.1 USD, much less than 24.83 ± 8.3 USD using insulin, which shows that *metformin* has the advantage of low price.

In this study, there was still heterogeneity in the *metformin* application group (blood glucose index), which may be related to the dynamic application adjustment of *metformin* in the study. Some patients failed to respond to oral *metformin* in regulating blood glucose and still needed insulin, which may bias the results. Although 10 included literatures were good, some literatures did not describe allocation concealment and blind method, which may cause implementation bias. Funnel plot showed possible publication bias, the number of included literatures was small, and the sample of participants was also small. The relevant studies still need to be supported by evidence from the study with higher quality RCT.

## Summary

The results of this meta-analysis showed that the application of oral hypoglycemic drug *metformin* in the blood glucose control of gestational diabetes can play a hypoglycemic effect equivalent to insulin, control the weight of pregnant women, reduce the rate of abortion and cesarean section, and improve pregnancy outcomes.

## Data Availability

The original contributions presented in the study are included in the article/supplementary material, further inquiries can be directed to the corresponding author/s.
